# The Institutional Library

**Published:** 1916-09-02

**Authors:** 


### THE INSTITUTIONAL LIBRARY.

### A DOCTOR'S HEALTH INSURANCE SCHEME.

**A Medical Service for the Genuinely Necessitous Classes of the Community and for Them Only.** By VIVIAN T. GREENYER, F.R.C.S., Vice-President and Chairman of Council of the National Medical Union. (Author: Edzell, New Church Road, Hove. Price 1s.)

THE present scheme, as the author's relation to the National Medical Union indicates, has been conceived to replace "the utter failure of the Medical Benefit Section of the National Health Insurance Acts 1911 and 1913." After a lengthy historical introduction on the "economic organisation of England from the Tudor period," and on the development of the Poor Law, inserted, unnecessarily, to provide a definition of the genuinely necessitous, which the author in practice regards as all with a wage below £1 a week, the scheme of relief is described, and illustrated by diagrams. Mr. Greenyer views the problem as one to be solved by the compulsory insurance of all receiving, and dependent upon, the minimum wage or less. This minimum wage is apparently £1 a week; but a great point is made of the necessity for including the dependants of the insured. He also insists that the employers should pay the entire premium, and urges that this plan will prove acceptable to them because the number of insured, when limited to the necessitous, will be greatly reduced. The classes to be excepted from this compulsory insurance include domestic servants, indoor and outdoor, teachers, nurses, entered apprentices, and bank clerks.

To deal with the insured when sick all existing institutions are to be invoked; the profession is to be paid for its services on their behalf; and a system of local units is to be formed for purposes of treatment. Each unit would comprise existing county institutions, a district medical officer, midwife, nurse, chauffeur, and ambulance, and a chemist. The district medical officers, who, it is urged, should be recruited from recently qualified men, and hold office for five years at a salary of £250, with 2,000 patients assigned to each of them, would send in-patients to the voluntary hospitals, to which a payment would be made. The payment suggested is the actual cost of the bed *plus* 1 per cent. The visiting staff would attend, as at present, free, since, as at present, they would be dealing with necessitous people. The scheme of administration includes a local medical committee composed of representatives of the local professions and institutions, and a central board, on which the divisions, the General Medical Council, and the Government would be represented. Statistics are given which place the number of necessitous persons, as defined above, and including their dependants, at rather more than six million. Other figures give the author's estimate of the expenditure as five and a half million for the first year, and the income (of which only the sum to be derived from the insurance premiums is given), £3,761,000.

Such is a brief summary of the scheme. At least it aims at giving the insured institutional treatment and at publicly recognising the work of the hospitals in providing this, which is more than the authors of the Insurance Act have done. But as compulsory insurance will never now, we believe, concern itself only with the "necessitous," it is more useful to summarise than to criticise this scheme. It has been put forward apparently on Mr. Greenyer's own initiative, and not in his official capacity as chairman of the National Medical Union.

**Surgery in War.** By Major A. J. HULL, F.R.C.S. (London: J. and A. Churchill. 1916. Pp. 390 Price 10s. 6d. net.)

IF every newly commissioned officer joining the Royal Army Medical Corps were advised to procure and read this handy volume, we are confident that the average surgical efficiency of those leaving civilian practice for service abroad would be very greatly increased. As an exposition of the experience gained by surgeons since the outbreak of war, when faced by wounds of a nature largely novel and unanticipated, Major Hull's book is useful, and on the whole sound rather than brilliant. It would be futile to expect universal agreement with his opinions on many topics, but the reviewer has to admit that there are more questions than he had expected to find on which his experience does not bear out that of the author of this book and of his coadjutors. There is also a tendency to sit on the fence with regard to important questions—*e.g.* on the treatment of secondary hæmorrhage, upon which the civilian temporarily commissioned needs especially clear and authoritative guidance.

Major Hull is a supporter of Sir Almroth Wright's views as to the treatment of wounds with hypertonic salt solutions, salt packings, and similar devices. Apparently the scepticism of many of the surgical specialists in the base hospitals concerning this treatment is shared by some at least of the collaborators in this book; thus Lieut.-Colonel Harrison uses some guarded language which gives the impression that he is by no means convinced. The sections on cranial surgery are, perhaps, the best in the book: this chapter is excellent throughout, and contains much which all officers in field ambulances and casualty clearing stations should read and take to heart. Dr. Greenfield does not appear to be much impressed with the operative treatment of abdominal wounds, as developed in the casualty clearing stations during the last twelve months; his indications for operation on these cases are distinctly conservative. The same contributor's chapter on the general condition of the wounded is very feeble indeed. The other associate-contributors have done their work well.

